# Fear in children: what is the importance of gender

**DOI:** 10.1192/j.eurpsy.2023.1505

**Published:** 2023-07-19

**Authors:** C. Pires-Lima, M. H. Figueiredo, V. Guedes

**Affiliations:** CINTESIS - Center for Health Technology and Services Research, Porto, Portugal

## Abstract

**Introduction:**

When we were children we all remember hearing expressions like: “Do not be afraid.”, “What are you afraid of?”, “You are strong, you are not afraid!”. This primary emotion is introduced in our development associated, most of the time, with a negative connotation. However, we know that fear, as a normal response to a real or imagined threat, is an integral part of human development (Sequeira, 2011).

The existence of research on fears is relevant for the definition of developmental patterns, characteristics such as frequency and intensity, but also for the identification of risk factors that may be at the genesis of the development of anxiety disorders (Ollendick, King & Murris, 2002).

**Objectives:**

In Portugal, studies on fears are scarce, contrary to what happens in other countries (Sequeira, 2011).

**Methods:**

The study sample consists of 121 students from the 1st cycle of basic education, 65 (53.7%) attending the 3rd year of schooling and 56 (46.3%) attending the 4th year of schooling, 66 ( 54.5%) were female and 55 (45.5%) were male, aged between 7 and 10 years old (M=8.5; SD=0.61).

For each child, an adult, parents or parental figures also participated in the study, most of which were the mother (89.3%) and the remaining participants were the father (7.4%), father and mother (1.7%), grandmother (0.8% ) and brother (0.8%).

**Results:**

Fears are more frequent in females than males.

**Conclusions:**

In general, anxiety disorders tend to be more prevalent in girls than in boys (Ollendick, King & Murris, 2002).

**Disclosure of Interest:**

None Declared

